# A move in response to starvation

**DOI:** 10.7554/eLife.69680

**Published:** 2021-06-01

**Authors:** Rebecca Martina Fausten, Maria Bohnert

**Affiliations:** Institute of Cell Dynamics and Imaging, and the Cells in Motion Interfaculty Centre, University of MünsterMünsterGermany

**Keywords:** lipid droplet, mevalonate, nucleus-vacuole junction, sterol-ester, HMG-CoA Reductase, *S. cerevisiae*

## Abstract

When a yeast cell runs out of fuel, it can increase the flux through a central metabolic pathway by simply changing the location of an enzyme.

**Related research article** Rogers S, Hariri H, Wood NE, Speer NO, Henne WM. 2021. Glucose restriction drives spatial reorganization of mevalonate metabolism. *eLife* **10**:e62591. doi: 10.7554/eLife.62591

No matter what you do for a living, organizing your workspace will help you to work efficiently. One way to do this is to have a place for everything – be it a piece of equipment in your lab or a file on your computer – but if you are lucky enough to have a job that involves performing a number of different tasks, it will help to have everything organized so that you can easily reconfigure your workspace for the task at hand.

Spatial organization is also important in eukaryotic cells, particularly when it comes to the positioning of structures called organelles that carry out specific tasks within each cell. Organelles often need to collaborate with one another to perform their roles, giving rise to contact sites. These are regions where the surfaces of different organelles are attached to each other by tether proteins (Scorrano et al., 2019). A prominent contact site can be found between the nuclear endoplasmic reticulum and the lysosome-like vacuole of *Saccharomyces cerevisiae*. This structure is known as the nucleus vacuole junction or NVJ (Pan et al., 2000). Now, in eLife, Mike Henne (University of Texas Southwestern Medical Center) and colleagues – including Sean Rogers as first author – report that *S. cerevisiae* can use the NVJ to spatially re-organize an important metabolic pathway called the mevalonate pathway (Rogers et al., 2021).

The researchers started by performing a visual screen in which they exposed *S. cerevisiae* cells to a metabolic challenge – the acute deprivation of glucose – and then used microscopy to track the spatial location of several fluorescently labeled enzymes. They found that an enzyme called HMG-CoA reductase, which is usually found in the membrane of the endoplasmic reticulum, responded to glucose deprivation by moving to the NVJ contact site (Figure 1). This enzyme catalyzes the rate-limiting step in the mevalonate pathway, which is involved in the synthesis of numerous important products, including the vital membrane lipid sterol and its storage form, sterol ester.

**Figure 1.**
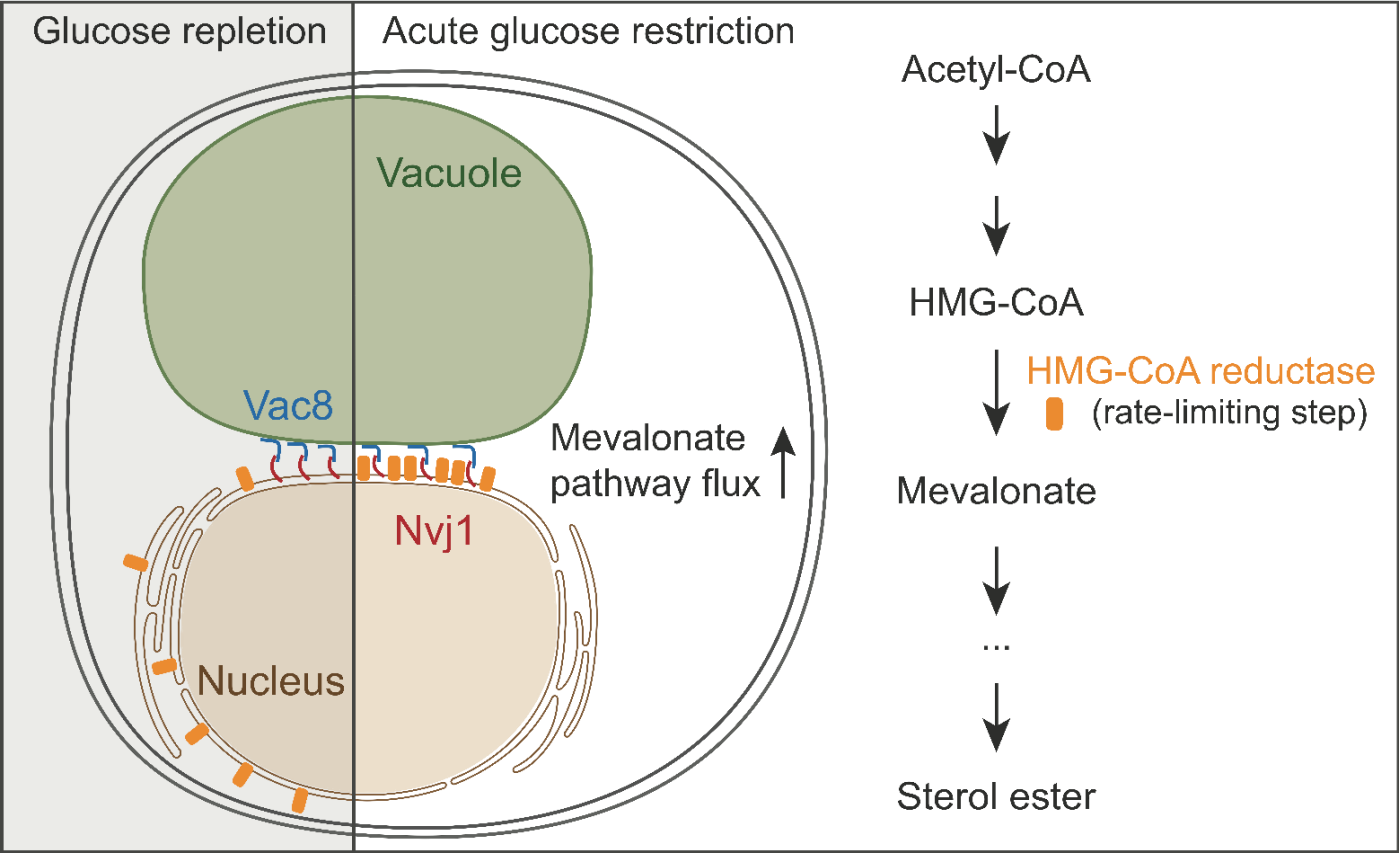
Enzyme partitioning in the mevalonate pathway. The enzyme HMG-CoA reductase (orange) is dispersed throughout the membrane of the endoplasmic reticulum (brown) in the presence of ample glucose (left), but it moves to a contact site between the cell nucleus and a lysosome-like organelle called the vacuole (green) in response to glucose deprivation (center). This nucleus-vacuole junction depends on two tether proteins, Vac8 (blue) and Nvj1 (red). The partitioning of HMG-CoA reductase to the contact site results in increased flux through the mevalonate pathway (right), and growth advantages once glucose becomes available again.

Rogers et al. also found that the rearrangement of the enzyme required a nuclear envelope protein called Nvj1 and a vacuolar protein called Vac8. These two proteins are responsible for contact site tethering between the nucleus and the vacuole, and the loss of either abolishes NVJ formation and partitioning of HMG-CoA reductase. In a beautiful structure-function analysis, Rogers et al. identified a mutant variant of Nvj1 that retained the ability to tether the nucleus to the vacuole, but lost the spatial compartmentalization of HMG-CoA reductase. This discovery allowed Rogers et al. to study the relevance of enzyme partitioning in metabolism independently of tethering loss.

Glucose starvation increases the flux of molecules through the mevalonate pathway, and Rogers et al. used the newly discovered Nvj1 mutant to show that this increase depends on the accumulation of HMG-CoA reductase at the NVJ. Furthermore, mutant cells unable to support enzyme partitioning had problems resuming growth once the supply of glucose was replenished. This defect could be rescued by supplementing the cells with mevalonate (the product of the reaction that HMG-CoA reductase catalyzes in the mevalonate pathway). Collectively, this shows that HMG-CoA reductase partitioning is beneficial for the cell.

But how can it be useful to move an enzyme to an organelle contact site? Other enzymes involved in sterol synthesis do not move to the NVJ, indicating that the enhanced flux is not a product of substrate channeling (enzymes passing intermediary products in a pathway directly to one another without releasing them into solution). Instead, Rogers et al. found that when several molecules of HMG-CoA reductase are in close proximity, the performance of the enzyme seems to improve. To demonstrate this in vivo, they generated a mutant version of HMG-CoA reductase fused to a fluorescent protein that forms tetramers to artificially force enzyme molecules to be in close proximity. In mutant cells unable to accumulate HMG-CoA reductase at the NVJ, expressing this enzyme variant had two effects: flux through the mevalonate pathway was restored, and growth was efficiently resumed once glucose was resupplied.

The NVJ is anything but a random site for placing a sterol metabolism enzyme. Ever since this contact site was discovered, many researchers have studied its role. Two decades of collaborative effort have revealed that the NVJ is involved in multiple processes related to the metabolism, transport, and storage of lipids (Kohlwein et al., 2001; Levine and Munro, 2001; Barbosa et al., 2015; Gatta et al., 2015; Lang et al., 2015; Murley et al., 2015; Hariri et al., 2018), and that it responds to metabolic stimuli by adjusting both its size and its protein composition (Bohnert, 2020). Thus, the study by Rogers et al. brings a new layer of complexity to our emerging view of the NVJ as a dynamic platform that coordinates the handling of lipids. In the future, untangling the interplay between the different lipid-related functions of this unique contact site may help us to understand how cells execute complex metabolic decisions.
